# The Relationship between Intimate Partner Violence, Rape and HIV amongst South African Men: A Cross-Sectional Study

**DOI:** 10.1371/journal.pone.0024256

**Published:** 2011-09-14

**Authors:** Rachel Jewkes, Yandisa Sikweyiya, Robert Morrell, Kristin Dunkle

**Affiliations:** 1 School of Public Health, University of the Witwatersrand, Johannesburg, South Africa; 2 Gender & Health Research Unit, Medical Research Council, University of the Witwatersrand, Pretoria, South Africa; 3 Research Office, University of Cape Town, Cape Town, South Africa; 4 Rollins School of Public Health, Emory University, Atlanta, Georgia, United States of America; Vanderbilt University, United States of America

## Abstract

**Objective:**

To investigate the associations between intimate partner violence, rape and HIV among South African men.

**Design:**

Cross-sectional study involving a randomly-selected sample of men.

**Methods:**

We tested hypotheses that perpetration of physical intimate partner violence and rape were associated with prevalent HIV infections in a cross-sectional household study of 1229 South African men aged 18–49. Violence perpetration was elicited in response to a questionnaire administered using an Audio-enhanced Personal Digital Assistant and blood samples were tested for HIV. A multivariable logistic regression model was built to identify factors associated with HIV.

**Results:**

18.3% of men had HIV. 29.6% (358/1211) of men disclosed rape perpetration, 5.2% (63/1208) rape in the past year and 30.7% (362/1180) of had been physically violent towards an intimate partner more than once. Overall rape perpetration was not associated with HIV. The model of factors associated with having HIV showed men under 25 years who had been physically violent towards partners were more likely to have HIV than men under 25 who had not (aOR 2.08 95% CI 1.07–4.06, p = 0.03). We failed to detect any association in older men.

**Conclusions:**

Perpetration of physical IPV is associated with HIV sero-prevalence in young men, after adjusting for other risk factors. This contributes to our understanding of why women who experience violence have a higher HIV prevalence. Rape perpetration was not associated, but the HIV prevalence among men who had raped was very high. HIV prevention in young men must seek to change ideals of masculinity in which male partner violence is rooted.

## Introduction

Gender-based violence and gender inequality are increasingly recognized as important structural drivers of the HIV epidemic in women [1], with research evidence from highly diverse cultural settings [2]–[5]. In attempts to explain this, authors have suggested that gender inequality and violence reduce women's ability to choose when and with whom to have sex and to protect themselves in sexual encounters. Other work suggests that men who are violent may be more likely to be HIV infected [2], [6]. Gender theory argues for the existence in many settings of masculinities that emphasise dominance and control of women, and legitimate use of violence to achieve and demonstrate this [7]–[9]. These ideas of masculinity are predicated on patriarchy and heterosexual success. Associated with them is a configuration of practice valorises having multiple sexual partners, and toughness or nonchalance related to risk, the latter often translates into non-condom use. In South Africa the hegemonic African masculinity is framed around such ideas of heterosexual success and control of women and is believed to provide the predominant underlying connection between men's use of violence against women and HIV [7], [8].

There is considerable evidence that men who are more violent and controlling engage more often in risky sexual practices and are more likely to have sexually transmitted infections [10]–[15]. But there is yet no published research showing associations between perpetration of rape and HIV sero-status, and no population-based study from a country with a generalized HIV epidemic on associations between HIV and IPV. Furthermore the limited evidence conflicts. In one study of young South African men the prevalence of perpetration of rape of a non-partner and of more than one episode of physical or sexual intimate partner violence was very similar among men with and without HIV (in each case 12% v. 16%) [16]. In contrast in India, married men were more likely to be HIV infected if they had perpetrated violence (aOR 1.91 95% CI 1.11, 3.27) [10]. Both these analyses, however, were based on very small numbers as the HIV prevalence was low, being 2% and 0.4% respectively.

Understanding the association between gender inequalities and gender-based violence and HIV, including the role of men's sero-status in these, is important for HIV prevention. Furthermore understanding whether men who rape may have a higher prevalence of HIV is critical for provision of post-rape care and ensuring that post-exposure prophylaxis for HIV is available [17]. To explore these issues in South Africa, a population based survey was conducted in two provinces with the aim of describing the prevalence and associations between rape, IPV perpetration and HIV sero-status among adult men and testing hypotheses that rape and IPV perpetration are associated with prevalent HIV infection.

## Methods

Ethics approval was given by the Medical Research Council's Ethics Committee. The study was undertaken in 2008 in three districts in the Eastern Cape and KwaZulu-Natal provinces of South Africa. These form a contiguous area, and include rural areas with communally-owned land under traditional leadership, as well as commercial farms, small towns, villages, and a city, inhabited by people of all South African racial groups, several ethnic groups (predominantly Xhosa and Zulu) and socio-economic backgrounds.

The sample used a two stage proportionate stratified design to identify a representative sample of men aged 18–49 years living in the three districts. Using the 2001 census as the primary sampling frame, 222 census enumeration areas (EAs) were selected as the primary sampling unit, stratified by district and with numbers proportionate to district population size. The sample was drawn by Statistics South Africa. Households in each EA were mapped and twenty were systematically selected. In each household one eligible man was randomly selected to take part in the interview. Men were eligible for the study if they were aged 18–49 years and had slept there the night before. Eligible men were asked to complete a questionnaire and provide a blood sample for HIV testing. This analysis includes only those who agreed to provide blood. There was no replacement.

Of the 222 selected EAs, two (0.9%) had no homes, and in five (2.3%) we could not interview because permission from the local political gatekeepers was declined (1) or we could not access any eligible home after multiple visits at different times of day (4). In all the latter EAs, we established that many households were ineligible due to age or absence of a man. We completed interviews in 215 of 220 eligible EAs (97.7%), and in these in 1,737 of 2,298 (75.6%) of the enumerated and eligible households. However only 70.8% (N = 1,229) of men interviewed provided blood i.e. 53.5% of enumerated and eligible men. Men who did not give blood did not differ in age or race from those who did, but they were more highly educated (47.9% has completed school v. 37.6% of those who gave blood) and less likely to have perpetrated physical IPV (25.0% v. 30.7% of those who gave blood).

Questionnaires were administered in isiXhosa or isiZulu and English using APDAs (Audio-enhanced Personal Digital Assistants) and took 45–60 minutes to complete. They included categorical variables measuring age, education, race, employment and income. Health questions asked about circumcision and history of a genital ulcer. Alcohol consumption in the past 12 months was assessed through a question on frequency of having 5 or more drinks per drinking day.

A 12-item scale assessed men's power and control in their main relationship with a female partner, after Pulerwitz et al [18], as adapted for South Africa by Dunkle et al [6]. These items were summed to derive a score (Cronbach's alpha 0.78). A typical question was “when I want sex I expect her to agree”. Men were asked about lifetime perpetration of physical intimate partner violence using the modified WHO violence against women instrument [19]. Specific acts of violence were asked about in five items ranging from slapping to threats with or use of a weapon. We have followed previous practice of categorising physical intimate partner violence into none or one episode versus more than one [2], [6], [11], [12].

We asked about frequency of condom use in the past year and about the number of primary and non-primary partners in the past year and lifetime. Concurrency was assessed by asking about having a *khwapheni* (an indigenous category of definitionally concurrent non-primary partner). We asked about transactional sex with women, defined as sex that was primarily motivated by a desire for material gain on the part of the woman. This was defined as providing food, cosmetics, clothes, transportation, items for children or family, school fees, somewhere to sleep, handyman work, or cash [6]. Interviewees were asked if they had ever had “sex with a prostitute”.

Rape of women and girls was assessed using seven questions adapted for the study from previously used items, and further validated through cognitive interviewing [20]. A typical item was “How many times have you slept with a woman or girl when she didn't consent to sex or after you forced her?”. Questions asked about rape of a current or ex-girlfriend or wife, rape of a non-partner, gang rape and rape of a woman who was drunk. Men were then asked two questions about perpetration of rape of a man or boy. We also asked about the age of the youngest victim raped.

The HIV tests were conducted on dried blood spots. These were tested with a screen ELISA (Genscreen, Bio-Rad, Steenvorde, France) and positive results confirmed with a second ELISA (Vironostika, bioMérieux, Marcy d'Etoile, France). They were analysed by the National Institute for Communicable Diseases, which participates in a programme supported by the Center for Communicable Diseases Control that has shown these methods can optimally identify HIV-1 from dried blood spots.

### Ethical issues

The men signed informed consent for the interview and separately for blood, if they agreed to a sample being taken. To recognise their research contribution, they were given R50 (US $6.5). The questionnaire asked men to disclose having engaged in a range of criminal acts. The nature of South African law meant that men could only be protected from possible legal repercussions through complete anonymity. To do this we had to keep no identifying information on interviewees, and this meant that we could not make return visits to offer HIV test results. HIV testing is free and widely available in South Africa and all men received a leaflet and were encouraged to go to their nearest clinic for HIV testing.

### Data analysis

Data were downloaded from the APDA memory cards and merged with the HIV test results. The resulting dataset provided a self-weighted sample. Analyses on participants who agreed to give blood (n = 1229) were carried out using Stata 10.0. All procedures took into account the two stage structure of the dataset, with stratification by district and the EAs as clusters. The social, demographic characteristics, sexual and violent practices and established HIV risk factors were summarised as percentages (or means) with 95% confidence limits, using standard methods for estimating confidence intervals from complex multistage sample surveys (Taylor linearization). Pearson's chi was used to test associations between categorical variables. No efforts were made to replace missing data.

To account for clustering of men within EAs, a random effects logistic regression model was fitted to identify factors associated with having HIV. Candidate variables for the model included social and demographic characteristics of the men, their sexual practices, relationship control, intimate partner violence (>1 episode v. none or one), any rape perpetration, substance abuse, having had anal sex, having had a genital ulcer and circumcision. The variables were entered into the model in groups and backwards elimination was used (p<0.2) to derive a final model. A term for stratum was included in the model. Variables for the final model were retained at p≤0.05. We tested for interactions between retained variables and found a significant interaction between perpetration of physical IPV and age; therefore present the association between physical IPV and HIV stratified by age of respondent.

## Results

**Figure 1:**
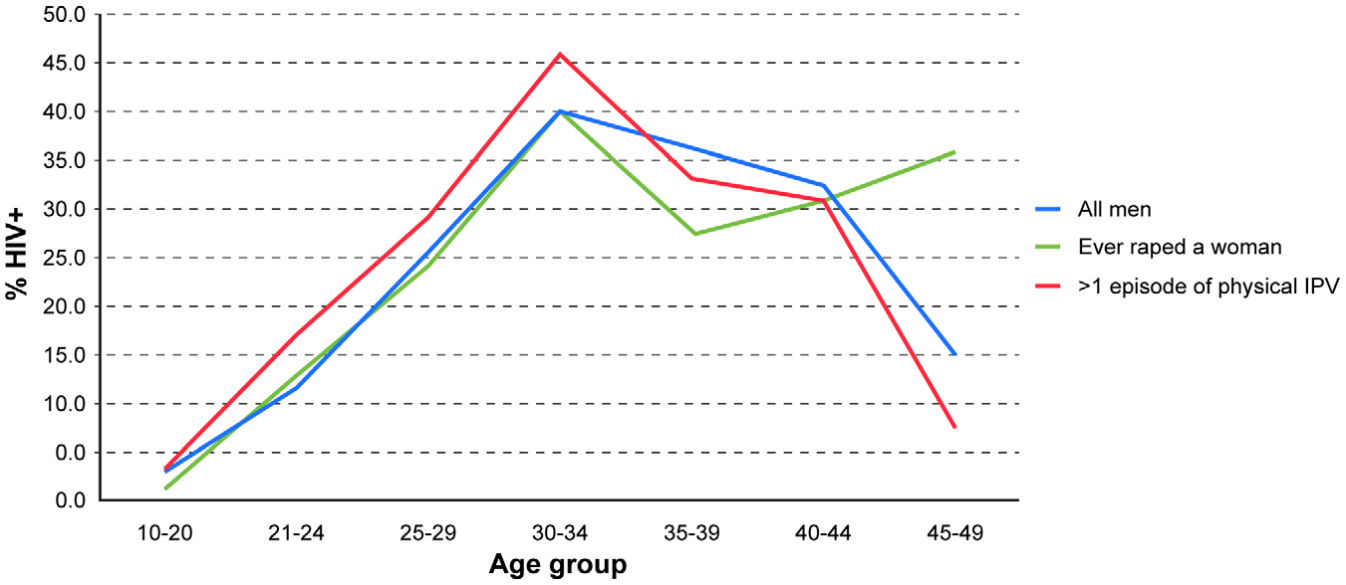
Age-specific HIV prevalence by perpetration of rape or physical intimate partner violence.

Overall 18.3% (225/1229) of men tested positive for HIV. Perpetration of rape of women was disclosed by 29.6% (358/1211) of men, 5.2% (63/1208) had raped in the past year and 30.7% (362/1180) of ever partnered men had been physically violent towards an intimate partner on more than one occasion. Figure 1 shows the age specific prevalence of HIV and a steep rise is seen to a peak in the 30–34 year age group, 40% of whom are infected. The figure shows that the age specific prevalence among men who have ever raped is very similar to that of all men. Men who have been physically violent towards a partner on multiple occasions have a higher age specific prevalence up to the 30–34 year age group and thereafter it is a little lower than that for all men. 95% confidence intervals are not presented in this figure, but for every data point they overlap.

The social and demographic characteristics of the whole sample and men with and without HIV are shown in Table 1. Most of the men in the study were Black African (85%), and more than half were under 25 years of age. Just over a third (38%) had completed high school or had some tertiary education, and only just over half (54%) had worked in the past year, and this was generally in low paid work as about 80% of those working earned under R2000 (US $270) per month. Men with HIV were older than other men and over half were aged 25–34 years. More of them were Black African (95%). Men with HIV were less well educated than those without HIV, and more were cohabiting, rather than married or single. There was little difference in income and the proportion working.

**Table 1:** Social and demographic characteristics of the sample.

	Total		HIV+		HIV−		
	n	%	n	%	n	%	p value
**Age (N = 1229):** 18–24 years	642	52.2	47	20.9	595	59.3	<0.0001
25–34 years	369	30.0	115	51.1	254	25.3	
35–49 years	218	17.7	63	28.0	155	15.4	
**Education: (N = 1224)**							
Primary or less	249	20.3	66	29.5	183	18.3	0.001
Some secondary	515	42.1	92	41.1	423	42.3	
School completion and higher education	460	37.6	66	29.5	394	39.4	
**Race (N = 1229):** Black African	1,040	84.6	214	95.1	826	82.3	<0.0001
Other	189	15.4	11	4.9	178	17.7	
**Marital status (N = 1229):** married	280	22.8	44	19.6	236	23.5	0.001
cohabiting with woman	145	11.8	44	19.6	101	10.1	
divorced/widower	45	3.7	9	4.0	36	3.6	
Single	759	61.8	128	56.9	631	62.9	
**Earned or worked in past 12 months (N = 1227)**	663	54.0	120	53.3	543	54.2	0.813
**Monthly income (N = 1139):** none	561	49.3	104	49.8	457	49.1	0.370
R1–R500	213	18.7	38	18.2	175	18.8	
R501–R2000	238	20.9	51	24.4	187	20.1	
R2001–R5000	76	6.7	9	4.3	67	7.2	
Over R5000	51	4.5	7	3.4	44	4.7	

High risk sexual practices were reported by a high proportion of all men (Table 2). However more men who were HIV positive had ever had transactional sex and sex with more than 20 partners, but a very similar proportion had consistently used a condom in the past year, drank alcohol heavily, had had sex with a prostitute/sex worker or had a khwapheni (concurrent partner). There was little difference in the proportion reporting ever having had anal sex with a man. More men with HIV had had a genital ulcer and fewer had been circumcised. There were some differences in relationship equity, with fewer men with HIV reporting having a relationship that was in the most equitable tertile.

**Table 2:** Sexual behaviours, violent practices and other potential HIV risk factors by HIV risks.

	Total		HIV+		HIV−		
	n	%	n	%	n	%	p value
Ever had transactional sex (N = 1194)	827	69.3	170	78.7	657	67.2	0.001
>20 lifetime partners (N = 1202)	417	34.7	88	40.6	329	33.4	0.058
Consistent condom use in the last year (N = 1065)	403	37.8	76	37.4	327	37.9	0.903
Heavy alcohol use in the last year (N = 1185)	215	18.1	32	15.0	183	18.8	0.223
Ever had sex with a prostitute (N = 1155)	213	18.1	41	19.3	172	17.8	0.603
Had a khwapheni in last year (N = 1189)	752	63.3	136	63.9	616	62.1	0.824
Ever had anal sex with a man (N = 1229)	54	4.4	13	5.8	41	4.1	0.283
Ever had a genital ulcer (N = 1182)	364	30.8	99	46.5	265	27.4	<0.0001
Ever been circumcised (N = 1182)	472	40.0	67	31.6	405	41.8	0.016
Relationship control scale: high equity (N = 1036)	179	17.3	19	9.8	160	19.0	0.003
mid equity	717	69.2	150	77.7	567	67.3	
low equity	140	13.5	24	12.4	116	13.8	
>1 episode of physical intimate partner violence (N = 1180)	362	30.7	86	39.3	276	28.7	0.003
Ever raped a woman (N = 1211)	358	29.6	70	31.3	288	29.2	0.533
Raped a woman in the past year (N = 1208)	63	5.2	8	3.6	55	5.6	0.223
Ever raped a man (N = 1205)	36	3.0	10	4.5	26	2.7	0.091
Ever raped a girl <15 (N = 1125)	60	5.3	10	5.0	50	5.4	0.796

Table 3 presents age-adjusted odds ratios for the association between HIV sero-positivity and perpetration of rape and physical intimate partner violence. Most of the measures of rape perpetration lacked evidence of association with HIV sero-status, however men who had been physically violent towards a partner were more likely to be HIV infected (aOR 1.50 95%CI 1.04, 2.17 p = 0.03).

**Table 3:** Association between perpetration of violence and HIV sero-positivity.

	HIV+ (n/N)	HIV− (n/N)	aOR*	95%CI		p value
Ever raped a woman	70/224	288/987	1.05	0.72	1.52	0.806
Ever sexually violent to an intimate partner	36/224	148/985	0.96	0.60	1.53	0.848
Ever rape a stranger or acquaintance	48/224	232/985	0.81	0.54	1.23	0.330
Ever raped a child <15	10/202	50/923	1.16	0.52	2.61	0.716
Ever gang raped	24/224	92/985	1.21	0.69	2.13	0.498
Ever raped a man	10/224	26/981	2.26	0.93	5.48	0.072
Raped in the past year	8/224	55/984	0.44	0.17	1.13	0.087
Physically violent to an intimate partner on >1 occasion	86/219	276/961	1.50	1.04	2.17	0.030

*adjusted for age group, circumcision and stratum.

Factors associated with having HIV are shown in Table 4. Being Black African and having ever had a genital ulcer were strongly associated with having HIV, and being circumcised was protective. Young men (under 25 years) who had been physically violent towards a female partner had twice the odds of having HIV as men of that age who had not been violent (aOR 2.05 95% CI 1.05–4.01, p = 0.034). There was no association between IPV perpetration and HIV among men 25 and older.

**Table 4:** Multivariable model showing factors associated with having HIV, including interaction between age and perpetration of physical IPV* (n = 1142).

	Among men under 25			Among men 25 and over		
	OR	(95% CI)	p	OR	95% CI	p
**Physical IPV**						
None or 1 episode	1.00	Ref		1.00		
>1 episode	2.08	(1.07, 4.06)	0.03	1.21	(0.78, 1.87)	0.39

	All men		
	OR	(95% CI)	p
**African**	4.92	(2.38, 10.16)	<0.0001
**Had a genital ulcer**	1.90	(1.32, (2.73)	0.001
**Circumcised**	0.48	(0.32, 0.74)	0.001

*adjusted for stratum.

## Discussion

Among adult South African men, HIV prevalence was significantly higher in men who had been repeatedly physically violent towards their partner compared to those who had not, however HIV prevalence was not elevated among men who had raped. After adjusting for other factors associated with HIV infection, being physically violent remained associated for younger men. Whilst this could have been a chance finding, the fact that it mirrors that of Maman et al among women in Tanzania, among whom the association between HIV and violence was seen in younger women [22], makes this less likely. Thus our hypotheses were partially supported by our data.

Whilst we cannot draw conclusions about temporality of associations, the findings of the association between physical partner violence against women and HIV sero-status are in line with the research which has found evidence of a clustering of men's violent and sexually risky practices [11]–[14]. Although similar clustering has also been found around the practice of rape perpetration [12]. Authors have argued that these practices have a common root in a unifying ideal of masculinity [8], and whilst this is highly plausible the interpretation of our findings on rape perpetration remain a challenge. Nonetheless changing this gender-inequitable ideal must be an important focus of HIV prevention programmes, because there is strong evidence that women in more gender inequitable relationships are at higher risk of HIV incident infections and our findings suggest that so doing will benefit to men and women alike [8]. The findings of the *Stepping Stones* evaluation in South Africa provide evidence that this can be done, male violence perpetration and risk taking can be reduced through jointly targeted intervention, with impact on sexually transmitted infections [21].

Not with standing the absence of association between perpetration of rape and HIV, our findings provide little comfort for rape survivors. The HIV prevalence among all men was very high, even if it was not elevated among those who had in the past or recently perpetrated rape. In South Africa post-exposure prophylaxis for HIV after rape is a very important part of post-rape care for survivors who are HIV negative.

The high HIV prevalence among men in the study was as expected in this population. In the country as a whole, for example, in 2008 the prevalence of HIV among African males aged 25–49 was 24% [23]. The high rates of disclosed perpetration of all forms of rape and partner violence was also comparable to findings of research with South African women [6] and men [12], [13]. The age structure of the men in the study differed a little from that found in all South African households in the 2003 South Africa Demographic & Health Survey, with fewer men aged 35–49 than the population (17% versus 33% in SADHS) [24]. Thus some caution is needed in generalising prevalences from the study to all men age 18–49 years in the population. Generalizability may be limited by the fact that the men who gave blood for HIV testing differed in some ways from those who refused, however adjusting the model for the socio-demographic variables that differed between those who did and did not test did not alter the findings.

A strength of the study was the use of APDAs for data collection, as these provides a confidential environment in which disclosure of anti-social and illegal behaviour could be enabled. Through removing the face to face component to interviews, APDAs greatly reduced the performative aspect of interview responses and so gave us more confidence that there would not have been a problem of over-reporting. Under-reporting, either deliberately, or through re-construing acts of force as those of persuasion, is a recognised problem in research on rape. It is impossible to estimate the magnitude of this in the study. Recall bias is a potential problem with any study of this nature. Self-completion of the questionnaire and the option of skipping questions resulted in some missing data on some items. We have not replaced missing values. We did not retain information on the number of eligible men per household and so were not able to weight the analysis for this, but we have not reason to believe this would have made much difference to the estimates of association [25].

This study provides further evidence of the need to engage with men to promote gender equity and reduce partner violence as part of HIV prevention. Whilst other studies have shown that gender inequities and violence are drives of the HIV epidemic in women [2], this study shows how harmful violent masculinities are for the health of men. These findings confirm the importance of engaging men to promote less violent and more respectful and gender equitable relations with women as part of the agenda of HIV prevention.
